# Repression of YdaS Toxin Is Mediated by Transcriptional Repressor RacR in the Cryptic *rac* Prophage of *Escherichia coli* K-12

**DOI:** 10.1128/mSphere.00392-17

**Published:** 2017-11-22

**Authors:** Revathy Krishnamurthi, Swagatha Ghosh, Supriya Khedkar, Aswin Sai Narain Seshasayee

**Affiliations:** a Shanmuga Arts, Science, Technology & Research Academy, Thanjavur, Tamil Nadu, India; b National Centre for Biological Sciences, Tata Institute of Fundamental Research, Bangalore, India; University of Iowa

**Keywords:** prophages, toxin-antitoxin, transcriptional repressor

## Abstract

Horizontal gene transfer is a major driving force behind the genomic diversity seen in prokaryotes. The cryptic *rac* prophage in *Escherichia coli* K-12 carries the gene for a putative transcription factor RacR, whose deletion is lethal. We have shown that the essentiality of *racR* in *E. coli* K-12 is attributed to its role in transcriptionally repressing toxin gene(s) called *ydaS* and *ydaT*, which are adjacent to and coded divergently to *racR*.

**IMPORTANCE** Transcription factors in the bacterium *E. coli* are rarely essential, and when they are essential, they are largely toxin-antitoxin systems. While studying transcription factors encoded in horizontally acquired regions in *E. coli*, we realized that the protein RacR, a putative transcription factor encoded by a gene on the *rac* prophage, is an essential protein. Here, using genetics, biochemistry, and bioinformatics, we show that its essentiality derives from its role as a transcriptional repressor of the *ydaS* and *ydaT* genes, whose products are toxic to the cell. Unlike type II toxin-antitoxin systems in which transcriptional regulation involves complexes of the toxin and antitoxin, repression by RacR is sufficient to keep *ydaS* transcriptionally silent.

## Abstract (precis)

Transcription factors in the bacterium *E. coli* are rarely essential, and when they are essential, they are largely toxin-antitoxin systems. While studying transcription factors encoded in horizontally acquired regions in *E. coli*, we realized that the protein RacR, a putative transcription factor encoded by a gene on the *rac* prophage, is an essential protein. Here, using genetics, biochemistry, and bioinformatics, we show that its essentiality derives from its role as a transcriptional repressor of the *ydaS* and *ydaT* genes, whose products are toxic to the cell. Unlike type II toxin-antitoxin systems in which transcriptional regulation involves complexes of the toxin and antitoxin, repression by RacR is sufficient to keep *ydaS* transcriptionally silent.

## INTRODUCTION

Horizontal gene transfer (HGT) contributes to the vast genome diversity seen in prokaryotes. The size of the genome of *Escherichia coli* ranges from under 4 Mb to under 6 Mb. The core genome constitutes only ~10% of the gene families represented across these *E. coli* genomes. The rest of the genetic content is variable across strains and often found in genomic islands (1). Many virulence factors and determinants of antibiotic resistance are known to be horizontally acquired and encoded, for example, by genes carried on autonomously replicating plasmids and chromosomally replicating prophages (2, 3).

The genome of the laboratory strain *E. coli* K-12 comprises nine cryptic prophages which constitute 3.6% of its total genome. Among these prophages is a cryptic prophage called *rac*. The *rac* prophage is 23 kb long and carries 29 genes. Among these genes is a putative transcription factor called RacR, whose deletion is lethal to the cell.

In attempting to explain the essentiality of the horizontally acquired *racR* gene, we used a combination of genetics, biochemistry, and bioinformatics to present evidence that RacR is indeed a transcriptional repressor. We show that RacR binds to its own upstream sequence and represses the adjacent and divergently coded *ydaS-ydaT* gene pair. *ydaS* and *ydaT* encode toxic products. Thus, the *ydaST-racR* module forms a “toxin-repressor” combination, making RacR essential to the cell.

## RESULTS

### RacR is an essential transcriptional regulator.

**FIG 1:**
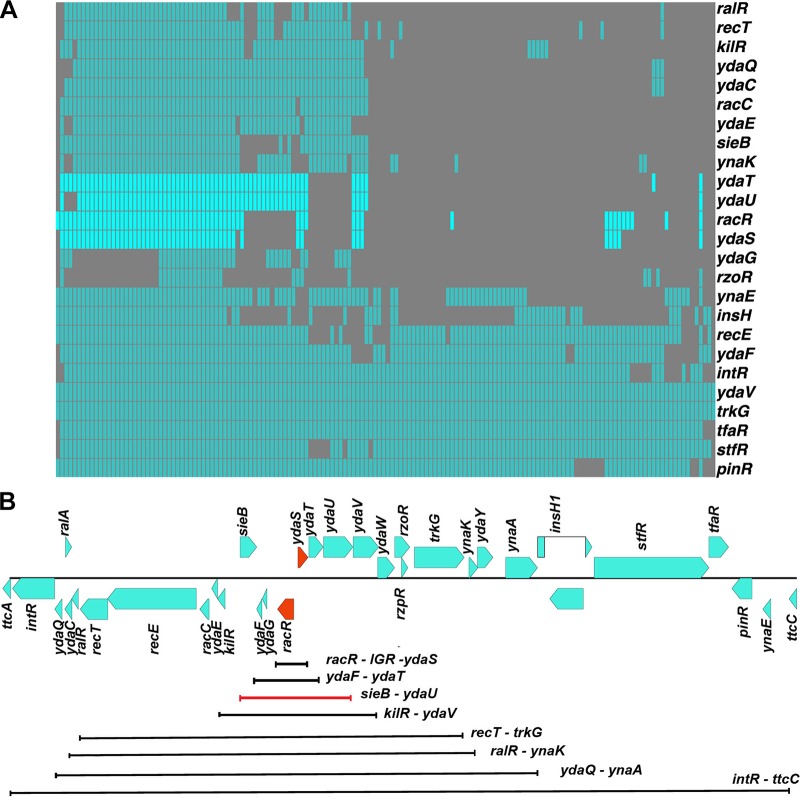
(A) Matrix showing the conservation of *rac* prophage genes across 154 *E. coli* genomes. The absence (gray) and presence (cyan) of any gene is indicated. The *racR*, *ydaS*, *ydaT*, and *ydaU* genes are shown in bright cyan. Note that the presence of *ydaS* is always accompanied by the presence of *racR*. (B) Map of the *rac* prophage showing the putative regulatory genes in orange. The lines indicate deletions, each of which included a deletion of *racR*. The red line shows the region from the *sieB* gene to the *ydaU* gene deleted, which left the *kilR* gene intact in the absence of* racR*.

The *rac* prophage is a cryptic and mosaic prophage in *E. coli*. Its size and gene content vary across *E. coli* strains, with only a few highly conserved genes, including *recE*, involved in an alternative homologous recombination pathway, and *trkG*, a potassium ion permease (Fig. 1A). Among the less-conserved portion of the *rac* prophage is a gene encoding a predicted transcription factor called RacR. It contains a weak helix-turn-helix motif and at best is very distantly related to the lambda *cI* repressor (15% identity by Needleman-Wunsch global alignment). Its deletion is presumed to be lethal. The Keio collection of *E. coli* single gene deletion mutants does not contain Δ*racR* (4), and we were unable to delete *racR* by homologous recombination. Nevertheless, the entire *rac* prophage could be deleted (we refer to this as Δ*rac* here), and the prophage excises at high rates in certain genetic backgrounds (5). Hence, we hypothesized that RacR could be a repressor of a toxin in the same prophage. Because the *rac* prophage carries a previously reported toxin called KilR, an inhibitor of cell division (6), we initiated our screen for the toxin by attempting to delete *racR* in the Δ*kilR* strain. However, we found that Δ*racR* could not be obtained even in a Δ*kilR* background.

We then deleted successively shorter segments, each containing *racR*, of the prophage. If a deletion attempt removed *racR* but not the toxin that RacR might repress, we would not recover the mutant. The smallest deletion we obtained by this approach included* racR*, its neighboring, divergent gene *ydaS*, and the common intergenic region (henceforth referred to as IGR) between them (Fig. 1B). Thus, the absence of *ydaS* and the common IGR between *racR* and *ydaS* is a suppressor of the lethality of Δ*racR*. Despite several attempts, we were unable to delete *racR* in the Δ*ydaS* mutant without disturbing the IGR between them. However, we obtained Δ*racR* with its IGR intact in a Δ*ydaS-T* (deletion of *ydaS* and *ydaT* and the short ~20-bp spacer separating the two ORFS) background. *ydaT* is encoded in tandem and downstream of *ydaS* and might be part of the same operon.

### Overexpression of *ydaS* and *ydaS-T* reduces growth.

**FIG 2:**
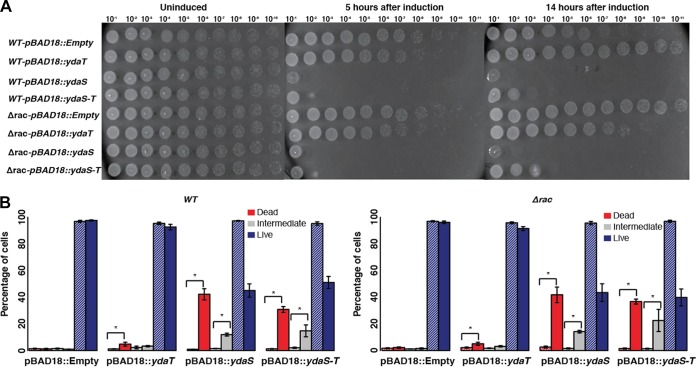
(A) Log- and stationary-phase cultures of pBAD18*-ydaS*, pBAD18*-ydaT*, pBAD18*-ydaS-T*, and empty vector in the wild-type (WT) and Δ*rac* backgrounds, grown in the presence or absence of 0.2% l-arabinose, were spotted onto the LB plate without arabinose. (B) Live/dead assay of pBAD18*-ydaS*, pBAD18*-ydaT*, pBAD18*-ydaS-T*, and empty vector in wild-type and Δ*rac* backgrounds. The cells were collected after 5 h of induction and treated with propidium iodide (PI) to mark the dead cells. The bar graphs show the percentages of dead, intermediate, and live population of cells in uninduced cultures (hatched bars) and induced cultures (solid bars). Values for the induced and uninduced constructs that are significantly different (*P*  < 0.01) by Wilcoxon rank sum test are indicated by a bar and asterisk. The error bars represent the standard errors computed from six independent trials (three biological replicates and two technical replicates).

We tested the toxicity of *ydaS*, *ydaT*, and *ydaS-T* by cloning these genes under the *araBAD* promoter in pBAD18. Expression of these cloned genes was induced in both the wild type and Δ*rac* mutant with 0.1% l-arabinose. We found that the expression of *ydaS* and *ydaS-T* causes rapid growth inhibition after induction in both the wild type and Δ*rac* mutant (see Fig. S1 in the supplemental material). We collected samples at 5 h and 14 h after induction and spotted these samples onto agar plates. Cells expressing *ydaS* and *ydaS-T* from pBAD18 did not grow on these plates (Fig. 2A). The expression of *ydaT* alone did not have any inhibitory or lethal effect on the wild type or the Δ*rac* prophage strain. However, we subsequently noticed that YdaT when expressed as described here accumulates in the insoluble fraction, and therefore might not be functional.

10.1128/mSphere.00392-17.1 FIG S1  Growth curve of the clones pBAD18*-ydaS*, pBAD18*-ydaT*, pBAD18*-ydaS-T*, and empty vector in wild-type and Δ*rac* backgrounds showing that induction of *ydaS* and *ydaS-T* in tandem reduces the growth rate irrespective of the strains used. Download FIG S1, TIF file, 1.2 MB. Copyright © 2017 Krishnamurthi et al. 2017 Krishnamurthi et al. This content is distributed under the terms of the Creative Commons Attribution 4.0 International license.

Further, we quantified the live- and dead-cell populations after the induction of *ydaS*, *ydaT*, and *ydaS-T* by fluorescence-activated cell sorting (FACS) using propidium iodide (PI) as a marker for dead cells. Results from six independent trials show that *ydaS* and *ydaS-T* expression, irrespective of the strain background, lead to loss of cell viability (Fig. 2B). We noticed that cells expressing *ydaS-T* were longer than the cells expressing *ydaS* or *ydaT* (Fig. S2).

10.1128/mSphere.00392-17.2 FIG S2  Bright-field images showing the increased cell size for the cells expressing *ydaS-T* compared to the cells expressing *ydaS* or *ydaT* alone. Bar, 20 µm. Download FIG S2, TIF file, 2.5 MB. Copyright © 2017 Krishnamurthi et al. 2017 Krishnamurthi et al. This content is distributed under the terms of the Creative Commons Attribution 4.0 International license.

Together, these results show that the expression of *ydaS* and *ydaS-T* is lethal and that *ydaS* and *ydaT* do not form a toxin-antitoxin (T-A) pair as predicted earlier (7). YdaS is critical to cell killing. While we could not successfully overexpress functional YdaT, the fact that we could isolate Δ*racR* only in a Δ*ydaS-T* background and not in a Δ*ydaS* background indicates that YdaT is also toxic to the cell. In a separate study, we show that clustered regularly interspaced short palindromic repeat (CRISPR)-Cas-mediated knockdown of *racR* causes a growth defect and also results in a filamentous cell phenotype in Δ*ydaS* cells and in Δ*ydaT* cells, but not in Δ*ydaS-T* cells (23). Another independent study by Campos and colleagues has shown that the Δ*ydaS* strain from the Keio collection shows a filamentous phenotype; this the authors attribute to a polar effect on the expression of *ydaT* (8). It is unclear why the overexpression of *ydaS*, despite being toxic, does not result in a filamentous phenotype in this study.

### Cooccurrence of *racR* and *ydaS* implies interaction between them.

Functionally related genes tend to be conserved together across genomes (9). We examined the conservation of genes of the *rac* prophage across 154 *E. coli* genomes. Bidirectional best-hit search for orthologs confirmed the mosaic nature of the *rac* prophage. In fact, more than 50% of the strains have lost half of the prophage. The genes that are well conserved across the genomes are the genes, such as *recE* and *trkG*, that have documented functions in the host. Some classical phage genes like *intR*, *pinR*, *stfR*, *tfaR*, *ydaF*, and *ydaV* are conserved in more than 85% of the strains analyzed.

We observe that the known toxin genes in the prophage are lost in most of the strains, and when they are present, they are always accompanied by its cognate antitoxin genes. RalR-RalA is a known type I T-A system in the same prophage (10). We observe that the RalR toxin is conserved in only 36.3% of the strains we analyzed; the corresponding noncoding antitoxin gene was found in all these strains. KilR, previously reported as a FtsZ inhibitor, was found in 48% of the strains in this analysis; its antitoxin, if any, is unknown. YdaS is present in only 33.7% of the strains analyzed, and we observe that it always cooccurs with RacR (Fig. 1A). A few strains carried the *ydaT* gene in the absence of *racR*; however, the IGR was lost in these strains, and certain point mutations were found in the *ydaT* gene. Thus, genome context analysis suggests a functional interaction between RacR and YdaS(-T).

### Expression of *ydaS* is kept silent under normal physiological conditions.

**FIG 3:**
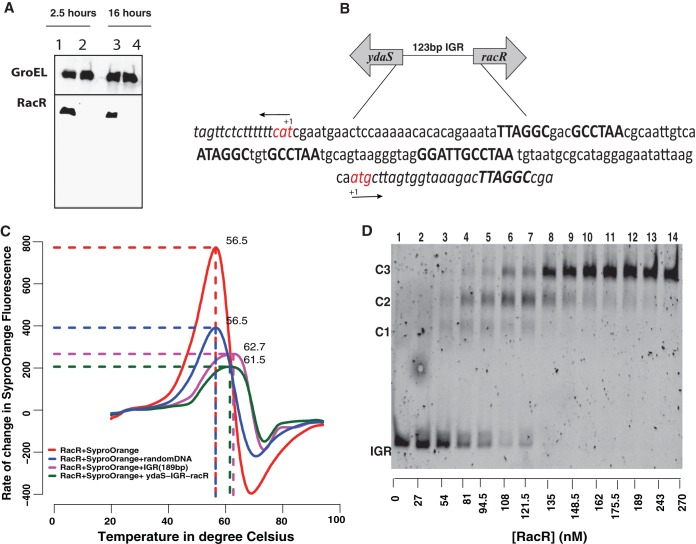
(A) Western blot showing the expression of RacR.YdaS expression could not be detected. The top blot contains GroEL as a loading control, and the bottom blot shows RacR expression during log and stationary phase. Twenty micrograms of total protein was used. (B) The intergenic region (IGR) between *ydaS* and *racR* showing the repeat elements with slightly different sequences in three different regions (boldface sequence). Note that the *ydaS* and *racR* are coded divergently and in opposite strands. The start codons of both genes are shown in red. (C) Thermal shift assay showing an ~5°C shift in the denaturation temperature (*T*_*D*_) of RacR in the presence of IGR. (D) Nonradioactive electrophoretic mobility shift assay showing the binding of RacR with IGR with distinct complexes marked as complex 1 (C1), C2, and C3. The 123-bp IGR (15 nM) was titrated against increasing concentrations of RacR from 27 nM to 270 nM (lanes 2 to 14). Lane 1 contains only 15 nM IGR without RacR.

In order to examine the expression of RacR and YdaS *in vivo*, we tagged these two genes with C-terminal 3×FLAG (DYKDDDDK). Western blotting using an anti-FLAG antibody showed that RacR was expressed throughout batch growth. However, YdaS expression could not be detected in our experimental conditions (Fig. 3A). An absolute protein quantification study performed by Li et al. (11) also shows a low copy number for YdaS. Analysis of various publicly available and in-house transcriptome sequencing (RNA-seq) data showed that the expression of *ydaS* is comparable to that of *bglG*, a well-characterized transcriptionally silent cryptic gene (12). *racR* was among the most highly expressed genes in the *rac* prophage, but only to a level comparable to that of the *lac* repressor gene (Fig. S3). These results show that YdaS is not expressed in *E. coli* and, in light of the genetic experiments reported above, lead to the hypothesis that RacR is a repressor of this toxin.

10.1128/mSphere.00392-17.3 FIG S3  Gene expression distribution of all the *rac* prophage genes is plotted as reads per kilobase of transcripts per million mapped reads (RPKM) from various RNA-seq data. *racR* is one of the few genes in the *rac* prophage found to be expressed over the conditions tested. *ydaS* and other toxin genes in the prophage are kept silent along with the *bgl* operon genes. Download FIG S3, TIF file, 1.5 MB. Copyright © 2017 Krishnamurthi et al. 2017 Krishnamurthi et al. This content is distributed under the terms of the Creative Commons Attribution 4.0 International license.

### Binding of RacR in the IGR.

RacR comprises a helix-turn-helix (HTH) motif, and hence, we investigated whether it binds to DNA. The 123-bp IGR between *racR* and *ydaS* contains three slightly variant repeats of GCCTAA and its inverse TTAGGC (Fig. 3B). This is similar to the regulatory region of lambda phage, which is bound by CI and Cro, even though the exact sequences bound by the proteins are different. To test for the binding of RacR to the IGR, we first performed a thermal shift assay with purified RacR and various nucleic acid sequences. The thermal shift assay measures the thermal denaturation temperature (*T*_*D*_) of a test protein. A change in this temperature in the presence of a ligand might argue in favor of an interaction between the protein and ligand. We found that the *T*_*D*_ of RacR increased by ~5°C in the presence of *racR*-IGR-*ydaS* or a 189-bp sequence upstream of *ydaS* and including the IGR (Fig. 3C). The extended 189-bp region, including a portion of the *racR* gene, was chosen for this experiment because this included an additional half-site of the above-mentioned palindrome.

We then performed a chromatin immunoprecipitation (ChIP) of RacR::3×FLAG to test for the binding of RacR to the IGR *in vivo*. By performing quantitative PCR (qPCR) against the DNA thus recovered, we found that the IGR was 2.5-fold enriched in comparison to a random region (Fig. S4A). Finally, we performed electrophoretic mobility shift assay (EMSA) to investigate the binding of purified RacR to the IGR. RacR formed three distinct complexes in the presence of the IGR (Fig. 3D). EMSA with a 49-bp DNA upstream of *ydaS*, containing a single copy of the repeat, also showed binding to RacR (Fig. S4B). Consistent with the view that the three palindromic repeats might be the sites to which RacR binds, we found only a single protein DNA complex with the 49-bp segment of the IGR. Thus, we show binding of RacR to the intergenic region between *racR* and *ydaS* both *in vitro* and *in vivo*.

10.1128/mSphere.00392-17.4 FIG S4  (A) ChIP qPCR showing the enrichment for IGR in the immunoprecipitated (IP'ed) DNA. Fold change was calculated by using 2^−ΔΔ*Ct*^ after normalizing the IP'ed and mock threshold cycle (*Ct*) to the input *Ct*. Results are shown for the qPCR done in triplicate for two biological replicates of IP'ed sample. Values that are significantly different (*P* < 0.01) by the Wilcoxon rank sum test are indicated by an asterisk. (B) EMSA showing the binding of RacR with a 49-bp region upstream of *ydaS*. The 49-bp DNA (23.5 nM) was titrated against increasing concentrations of RacR until 67.5 nM (lanes 2 to 6). Lane 1 contains the 49-bp region without RacR. Download FIG S4, TIF file, 0.6 MB. Copyright © 2017 Krishnamurthi et al. 2017 Krishnamurthi et al. This content is distributed under the terms of the Creative Commons Attribution 4.0 International license.

### Transcriptional repression of *ydaS* is mediated by RacR binding to the IGR.

**FIG 4:**
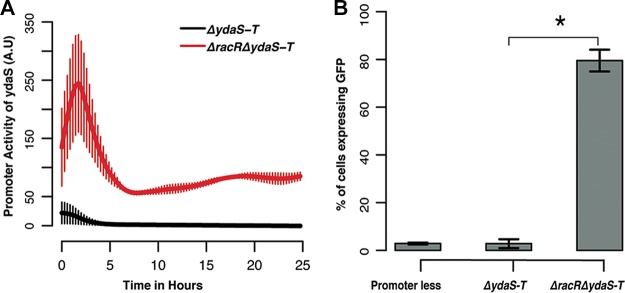
(A) IGR cloned in the low-copy pUA66 plasmid, showing promoter activity (in arbitrary units [A.U]) of *ydaS* in Δ*ydaS-T* and Δ*racR* Δ*ydaS-T* strains. (B) Bar graph representing the percentage of cells showing *ydaS* promoter activity. Values that are significantly different (*P* < 0.01) by *t* test are indicated by a bar and asterisk. The error bars represent the standard deviations computed from three independent trials.

Finally, to test whether the binding of RacR represses *ydaS*, we cloned the IGR upstream of *gfp-mut2* in pUA66. We monitored the promoter activity of pUA66::IGR-*gfp-mut2* in Δ*ydaS-T* cells and in Δ*racR* Δ*ydaS-T* cells for 25 h. We observed that the *ydaS* promoter is active only in Δ*racR* Δ*ydaS-T* cells; no fluorescence from *gfp-mut2* could be detected in Δ*ydaS-T* cells (Fig. 4A). The maximal *ydaS* promoter activity was observed in the log phase (optical density at 600 nm [OD_600_] of ~0.2 to 0.3). We tested the expression of *gfp-mut2* from these strains grown to mid-exponential phase using FACS. The distribution of fluorescence from pUA66::IGR-*gfp-mut2* in Δ*ydaS-T* cells was similar to that of the promoterless control where most cells were green fluorescent protein (GFP) negative. In contrast, in Δ*racR* Δ*ydaS-T* cells, nearly 80% of the cells were GFP positive (Fig. 4B). Thus, single-copy availability of RacR from the chromosome appears to be sufficient to suppress the activity of the *ydaS* promoter from a multicopy (three or four) plasmid. Thus, RacR represses transcription of *ydaS*.

## DISCUSSION

We have shown that the expression of *ydaS* and *ydaS-T* is lethal, and we attribute the essentiality of *racR* to its role in repressing the expression of this toxin. Earlier studies have shown the presence of two toxins, KilR and RalR, in the *rac* prophage (6, 10). In the present work, we suggest that that YdaS-YdaT (YdaS-T) is yet another toxin(s) encoded by the *rac* prophage. We do not know how this toxin effects cell killing and whether other genes in the operon to which *ydaS* and *ydaT* belong contribute to cell killing. The YdaS-T module could be compared to the orphan toxin OrtT for which the antitoxin counterpart is not known (13). In general, essential transcription factors are rare in *E. coli*. The essentiality of RacR is purely by virtue of its role in keeping a toxin transcriptionally silent. RacR is unlikely to have too many additional targets, because its expression level, based on RNA-seq, is very similar to that of the highly specific Lac repressor. Among the few essential transcription factors in *E. coli* is the antitoxin MazE. *mazE* and *mazF* are carried on the same operon, unlike *racR-ydaS*, which make a divergent gene pair. The antitoxin activity of MazE is primarily by protein-protein interactions with the toxin MazF. In fact, the binding of MazE to DNA is enhanced when in complex with MazF (14). Yet another essential transcription factor is the antitoxin MqsA, which again sequesters its cognate toxin MqsR. Unlike the conditional cooperativity displayed by MazE and MazF in binding to the DNA, the high stability of the MqsR-MqsA (MqsR-A) complex makes the protein-protein interaction mutually exclusive of MqsA-DNA interactions (15). In both these cases, it is apparent that the activity of the transcriptional repressor does not entirely prevent the expression of the toxin. In our case, however, we could not detect the presence of the YdaS protein, the expression level of the *ydaS* transcript is comparable to that of a bona fide cryptic gene across tens of RNA-seq data sets, and we cannot detect any activity from the *ydaS* promoter fused to *gfp-mut2* in the presence of RacR. The expression of YdaS-T is toxic, independent of the presence of RacR (wild type versus Δ*rac* mutant), which argues against the possibility of RacR interacting physically with YdaS/YdaT in suppressing its activity.

RacR is a repressor of *ydaS-T*, and this module is an example of a “toxin-repressor” system, where the toxicity of YdaS is repressed totally at the transcriptional level. The fact that the activity of the toxin is totally suppressed at the level of transcription initiation itself might render postsegregational killing downstream of the loss of the module impossible.

We propose that RacR could be functionally similar to the CI repressor of lambda prophage. The *rac* prophage has lost many of its structural genes compared to the lambda phage (see Fig. S5 in the supplemental material). However, the organization of regulatory elements in the *rac* prophage (Fig. 3B) is similar to the cI-Cro switch of lambda prophage (16). There are three repeat elements in the IGR, which might be the operator of this prophage. Our observation of the formation of three distinct DNA-protein complexes of the 123-bp IGR with increasing concentrations of RacR suggests that the IGR might act as a complex regulatory switch that resembles the regulatory region of *cI-cro* of lambdoid phages (17).

10.1128/mSphere.00392-17.5 FIG S5  Comparison of the *rac* prophage with the lambda prophage. The *rac* prophage has lost most of its structural genes compared to the lambda prophage. The other known toxins of *rac* prophage are shown in red. The regulatory genes in both prophages are shown in orange. Easyfig was used to generate the map of both prophages. Download FIG S5, PDF file, 0.3 MB. Copyright © 2017 Krishnamurthi et al. 2017 Krishnamurthi et al. This content is distributed under the terms of the Creative Commons Attribution 4.0 International license.

## MATERIALS AND METHODS

### Media, strains, and plasmid construction.

*E. coli* K-12 MG1655 from CGSC was used and grown at 37°C in Luria broth (LB) or LB agar (HiMedia). The antibiotic-resistant strains were grown in antibiotics wherever required; ampicillin (100 µg/ml), kanamycin (50 µg/ml), or chloramphenicol (30 µg/ml) was used. All the knockout strains were constructed by the one-step inactivation method described by Datsenko and Wanner using pKD13 as the template plasmid for the kanamycin resistance cassette amplification (18). Tagging of *racR* with 3×FLAG at the C-terminal end was done using the pSUB11 plasmid (19). Ectopic expression of *racR*, *ydaS*, *ydaT*, and *ydaST* were achieved by cloning them between the EcoRI and SalI sites of pBAD18; this brings the genes under the arabinose-inducible *araBAD* promoter. The plasmid for the promoter activity was constructed by cloning the intergenic region (IGR) in the low-copy-number vector pUA66 between the XhoI and BamHI sites. The strains and plasmids used in the current study are given in Table S1 in the supplemental material, and the primers used for gene deletion and cloning are listed in Table S2.

10.1128/mSphere.00392-17.6 TABLE S1  Strains and plasmids used in this study. Download TABLE S1, PDF file, 0.1 MB. Copyright © 2017 Krishnamurthi et al. 2017 Krishnamurthi et al. This content is distributed under the terms of the Creative Commons Attribution 4.0 International license.

10.1128/mSphere.00392-17.7 TABLE S2  Primers designed for gene deletion and cloning. Download TABLE S2, PDF file, 0.1 MB. Copyright © 2017 Krishnamurthi et al. 2017 Krishnamurthi et al. This content is distributed under the terms of the Creative Commons Attribution 4.0 International license.

### Growth curve and spotting assay.

Growth curve was monitored in a 96-well plate with the final volume of 200 µl using a Tecan F200 reader. Overnight culture was inoculated in the ratio of 1:100 and allowed to grow until an optical density at 600 nm (OD_600_) of 0.4 was reached. This was further diluted in fresh medium to an OD_600_ of 0.01 with or without 0.1% l-arabinose, and *A*_600_ was recorded for 14 h. For the spotting assay, appropriate overnight cultures were inoculated in LB broth containing 100 µg/ml ampicillin (diluted 1:100) with or without 0.2% l-arabinose. The cells were collected after 5 h and 14 h of inoculation, serially diluted, and spotted on LB agar plates containing ampicillin without arabinose.

### FACS.

Overnight cultures of the respective strains were inoculated in LB broth at 1:100 dilution with or without 0.2% l-arabinose. Samples were collected after 5 h of induction, pelleted, washed, and resuspended in 500 µl of saline (0.9% sodium chloride [wt/vol]). Exponentially growing cells were used as a live-cell control, and cells subjected to 80°C for 10 min were used as a dead-cell control. Propidium iodide (PI) solution (5 µl of a 1-mg/ml solution) was added to all the vials 10 min before acquisition of data in a BD FACSCalibur. Around 20,000 cells were acquired for each sample using a 488-nm excitation laser, and the emission was recorded from FL2 channel that uses a 585/42 band-pass (BP) filter to collect the PI intensity. The intermediate population in this study is described as the cells that fall between the region of live unstained control and dead control.

Exponential culture of Δ*ydaS-T* and Δ*racR* Δ*ydaS-T* bacteria containing pUA66::IGR-*gfp-mut2* were pelleted, washed, and resuspended in saline. Green fluorescent protein (GFP) intensity was monitored using FL1 channel that uses a 530/30 BP filter. A strain containing empty pUA66::*gfp-mut2* was used to set the background fluorescence, and GFP intensity above this background was marked as positive. Data were analyzed using Flowing software (Cell Imaging Core of the Turku Centre for Biotechnology [http://www.flowingsoftware.com/]).

### Bidirectional search for orthologous genes.

The genomes of 154 completely sequenced *E. coli* strains were downloaded from NCBI refseq ftp site. A bidirectional search for orthologous genes of the *rac* prophage, excluding pseudogenes, was performed using phmmer (version 3.1). The E-value threshold used was 10^−20^. An ortholog presence-absence matrix was hierarchically clustered based on Euclidean distance with centroid linkage. Clustering was performed using Cluster 3 (http://bonsai.hgc.jp/~mdehoon/software/cluster/), and the heat map was generated using matrix2png (http://www.chibi.ubc.ca/matrix2png/).

### RNA-seq data analysis.

Raw reads from 15 different transcriptome sequencing (RNA-seq) studies (with a total of 61 fastq files) were obtained either in-house or from the NCBI GEO or EBI array express databases (Table S3). The SRA files from GEO were converted to fastq using fastq dump. Reads from the fastq file were aligned to NC_000913.3 genome using bwa. The aligned files were sorted using sam (sequence alignment/map) tools. Further, these sam files were used to get read counts per nucleotide, from which read counts per gene was generated. RPKM (reads per kilobase of transcript per million mapped reads) was calculated by normalizing the raw read counts to the length of the gene and further by the total number of mapped reads for each fastq file. The distribution of RPKM values of the *rac* prophage genes was plotted as a boxplot, along with those of the *bgl* operon genes and *lacI* as reference genes. Because differential expression was not a goal of this study, more state-of-the-art normalization methods such as those used by EdgeR or DEseq were not required.

10.1128/mSphere.00392-17.8 TABLE S3  Accession numbers of RNA-seq data used for calculating RPKM. Download TABLE S3, PDF file, 0.1 MB. Copyright © 2017 Krishnamurthi et al. 2017 Krishnamurthi et al. This content is distributed under the terms of the Creative Commons Attribution 4.0 International license.

### Western blotting.

Total protein from *E. coli* K-12 cells was prepared and quantified using a bicinchoninic acid (BCA) assay, and 20 µg of total protein was loaded in a 15% SDS-polyacrylamide gel. The gel was subjected to electrophoresis at 120 V for 1 h, and proteins were transferred to a nitrocellulose membrane. Monoclonal anti-FLAG antibody (Sigma) was used to bind the specific protein to which the FLAG is tagged, and the signal was detected using horseradish peroxidase (HRP)-conjugated anti-mouse IgG. HRP luminescence was further detected by West Dura reagent (Thermo Scientific). Digital images of the blots were obtained using an LAS-3000 Fuji imager.

### Chromatin immunoprecipitation.

Immunoprecipitation was performed by the method of Kahramanoglou et al. (20) except that cell lysis and DNA shearing were coupled together using a Bioruptor (Diagenode) with 35 cycles (1 cycle consisting of 30 s on and 30 s off) at a high setting. Immunoprecipitated samples were quantified with specific primers for the 123-bp intergenic region and a random primer (*wza*), which is not part of the *rac* prophage, using quantitative PCR. The fold enrichment was calculated using 2^−ΔΔ*Ct*^by the method of Mukhopadhyay et al. (21).

### RacR purification.

RacR was cloned between the NdeI and XhoI restriction sites in a pET28a expression vector with the C-terminal His tag. After confirmation of its sequence and orientation, this plasmid was transformed in the expression strain C41(DE3). A single colony of the C41 strain containing the pET28a::*racR* plasmid was inoculated in 5 ml LB containing 100 µg/ml ampicillin. This overnight culture was diluted to a 1:100 ratio in 10 ml of fresh LB for raising the secondary inoculum. When the secondary culture reached an OD_600_ of 0.4, it was seeded into 1 liter of fresh LB in a 3-liter baffled flask at 37°C. When the culture reached an OD_600_ of 0.6, RacR expression was induced by adding isopropyl-β-d-thiogalactopyranoside (IPTG) at a final concentration of 100 µM, and the flask was incubated at 25°C for 5 h. The culture was harvested, and the cells were resuspended in 100 ml of lysis buffer (50 mM Tris [pH 8.5], 500 mM NaCl, 5% glycerol, 1% NP-40, 1× Sigma protease inhibitor cocktail). The resuspended cells were sonicated for 30 cycles (1 cycle consists of 30 s on and 30 s off). Further, the lysate was passed through equilibrated 1-ml prepacked Histrap column (Invitrogen) at a flow rate of 0.5 ml/min. The column was then washed with 50 ml of elution buffer (50 mM Tris [pH 8.5], 500 mM NaCl, 5% glycerol) containing 10 mM imidazole and then with 20 ml of elution buffer containing 50 mM imidazole and 100 mM imidazole. Finally, RacR was eluted with 10 ml of elution buffer containing 250 mM imidazole. Purified RacR was further passed through a Superdex 200 10/300 size exclusion column, which was preequilibrated with the same elution buffer without imidazole.

### Thermal shift assay.

DNA (0.3 µM) (*ydaS* with 189 bp upstream of it, including a portion of *racR*, *racR-*IGR-*ydaS* or random DNA) was mixed with 3 µM purified RacR in the presence of 20× Sypro orange (Sigma-Aldrich), and the final volume of the reaction mixture was adjusted to 20 µl with RacR elution buffer. Three replicates of each sample were loaded in a 384-well plate and sealed with an optical adhesive cover. The fluorescence spectrum in the 635-nm–640-nm bin was recorded using ABI Via7 PCR with the standard melt curve experiment setting in which the temperature ranged from 20°C to 95°C at the rate of 1°C per min. The denaturation temperature (*T*_*D*_) was reported as the temperature at which the maximum *dF*/*dT* was recorded, where *dF*/*dT* is the rate of change in Sypro orange fluorescence (*F*) with respect to the temperature (*T*). The data were processed and plotted using a custom R script to calculate *dF*/*dT*.

### Electrophoretic mobility shift assay.

The entire 123-bp IGR was PCR amplified and gel purified. A 6% polyacrylamide gel was prepared from 40% acrylamide-bisacrylamide (80:1) stock and allowed to polymerize for 2 h. The gel was prerun for 30 min at 70 V, and the wells were washed before sample loading. DNA (20 nM) was mixed with increasing concentrations of RacR in 10× binding buffer (100 mM Tris buffer [pH 8], 10 mM EDTA, 1 M NaCl, 1 mM dithiothreitol [DTT], 50% glycerol, 0.1 mg/ml bovine serum albumin [BSA]) in a final volume of 20 µl in 0.2-ml PCR tubes. These tubes were incubated at room temperature for 1 h. After incubation, samples were mixed with 2.2 µl of 10× loading dye (10 mM Tris [pH 8], 1 mM EDTA, 50% glycerol, 0.001% bromophenol blue, 0.001% xylene cyanol) and run at 70 V in room temperature for 90 min. The gel was stained using SYBR green (Thermo Scientific) for 15 min. The stained gel was washed in distilled water twice and imaged using a Lab India gel doc system.

### Promoter activity.

The promoter activity of the *ydaS* IGR was monitored by transforming the pUA66::IGR-*gfp-mut2* construct in Δ*ydaS-T* and Δ*racR* Δ*ydaS-T* mutants. M9 medium with 0.2% glucose was used to culture the strains. Overnight culture containing the plasmid in the respective background strain was inoculated at a ratio of 1:100 in a 96-well flat transparent black plate (Corning) in wells in a total volume of 200 µl. The optical density (OD_600_) and the GFP intensity (excitation at 485 nm and emission at 510 nm) were measured using the Tecan multimode reader at every 16-min interval with continuous shaking in between at 37°C. The background optical density is subtracted by using the optical density obtained from the blank well. The background fluorescence intensity was subtracted by using the intensity obtained from the strain that has the promoterless empty vector. Promoter activity (PA) was calculated as the rate of change in the GFP intensity normalized by the average OD for the given time point as follows: PA = smoothed dGFP/*dt*/smoothed (OD_600_) (22). Data processing and analysis were done using custom R script.

### Accession number(s).

RNA-seq data have been deposited in the GEO database under accession number GSE104504. Additional accession numbers are listed in Table S3 in the supplemental material.
