# Supplementary figures and images for: Repression of YdaS Toxin Is Mediated by Transcriptional Repressor RacR in the Cryptic rac Prophage of Escherichia coli K-12
Source: mSphere. 2017 Nov 22;2(6):e00392-17. doi: 10.1128/mSphere.00392-17 (PMC5700373; doi:10.1128/mSphere.00392-17)

**A. cryptic rac prophage**

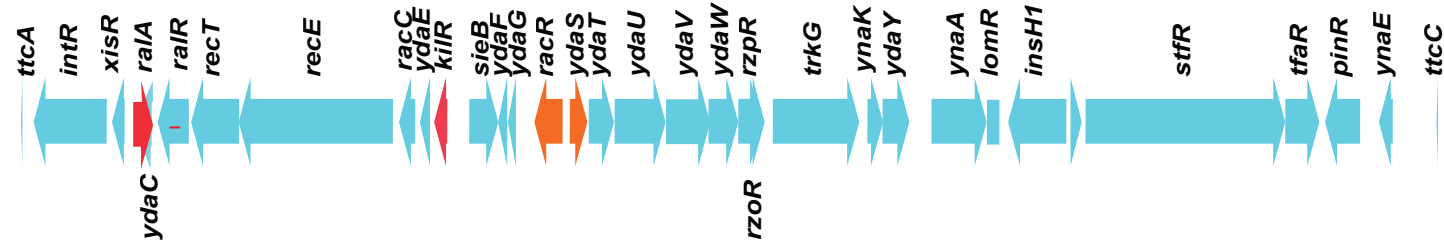

**B.  $\lambda$  phage**

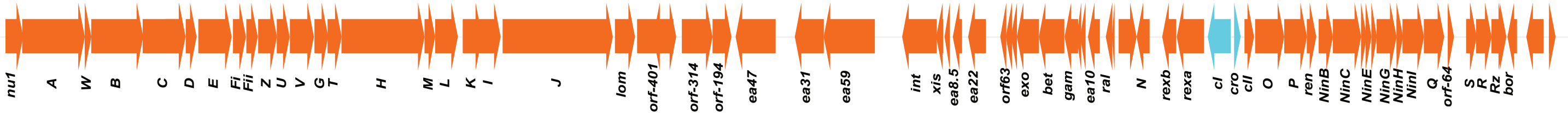

Supplement: FIG S5 [file sph005172392sf5.pdf]
