# Supplementary material for: Repression of YdaS Toxin Is Mediated by Transcriptional Repressor RacR in the Cryptic rac Prophage of Escherichia coli K-12
Source: mSphere. 2017 Nov 22;2(6):e00392-17. doi: 10.1128/mSphere.00392-17 (PMC5700373; doi:10.1128/mSphere.00392-17)
Supplement: TABLE S1 [file sph005172392st6.pdf]

Supplementary Table1: Strains and plasmids used in this study

**Strain Name**	**Description**	**Source**
MG1655	F-, λ^-, *rph-1*	CGSC
ΔydaF-ΔydaT	MG1655 Δ1419156-1421106 (*ydaF-ydaT*)	This study
ΔsieB-ΔydaU	MG1655 Δ1418671-1421976 (*sieB-ydaU*)	This study
ΔkilR-ΔydaV	MG1655 Δ1418008-1422729 (*kilR-ydaV*)	This study
ΔrecT-ΔtrkG	MG1655 Δ1413984-1425239 (*recT-trkG*)	This study
ΔralR-ΔynaK	MG1655 Δ1413733-1425640 (*ralR-ynaK*)	This study
ΔydaQ-ΔynaA	MG1655 Δ1413237-1427386 (*ydaQ-ynaA*)	This study
ΔintR-ΔttcC	MG1655 Δ1411925-1434984 (*intR-ttcC* entire rac prophage)	This study
ΔracR-ΔydaS	MG1655 Δ1420241-1420661 (*racR-ydaS* including the 123bp common IGR)	This study
ΔydaS-T	MG1655 Δ1420365-1421106 (*ydaS-ydaT*)	This study
ΔracRΔydaS-T	MG1655 Δ*ydaS, ΔydaT, ΔracR*	This study
ΔkilR	MG1655 *Δ kilR*	This study
Δ ydaS	MG1655 *Δ ydaS*	This study
Δ ydaT	MG1655 *Δ ydaT*	This study
Δ ydaF	MG1655 *Δ ydaF*	This study
Δ ydaG	MG1655 *Δ ydaG*	This study
racR::3XFLAG	MG1655 *racR::3XFLAG*	This study
ydaS::3XFLAG	MG1655 *ydaS::3XFLAG*	This study
C41(DE3)	OverExpress: F – ompT hsdSB (rB- mB-) gal dcm (DE3)	Lucigen
**Plasmid**	**Description**	**Source**
pBAD18	Arabinose Inducible vector	Gillian's lab
pBAD18::*racR*	pBAD18 carrying *racR*	This study
pBAD18::*ydaF*	pBAD18 carrying *ydaF*	This study
pBAD18::*ydaG*	pBAD18 carrying *ydaG*	This study
pBAD18::*ydaS*	pBAD18 carrying *ydaS*	This study
pBAD18::*ydaT*	pBAD18 carrying *ydaT*	This study
pBAD18::*ydaS-T*	pBAD18 carrying *ydaS* and *ydaT* in tandem	This study
pET28a	Overexpression Vector with C-Terminal His Tag	Novogen
pET28a::*racR*	pET28a vector carrying *racR* gene with C-Terminal HisTag	This Study
pUA66	Low copy plasmid with fast folding GFP mut2	SAFS lab
pUA66::IGR	pUA66 vector carrying 123 bp ydaS promoter	This study
pKD13	F-, *Δ(araD-araB)567*, *ΔlacZ4787*(::rrnB-3), *Δ(phoB-phoR)580*, λ^-, *galU95*, *ΔuidA3::*pir^+, *recA1*, *endA9*(del-ins)*::FRT*, *rph-1*, *Δ(rhaD-rhaB)568*, *hsdR514*, pKD13	CGSC
pKD3	F-, *Δ(araD-araB)567*, *ΔlacZ4787*(::rrnB-3), *Δ(phoB-phoR)580*, λ^-, *galU95*, *ΔuidA3::*pir^+, *recA1*, *endA9*(del-ins)*::FRT*, *rph-1*, *Δ(rhaD-rhaB)568*, *hsdR514*, pKD3	CGSC
pKD46	F-, *Δ(araD-araB)567*, *ΔlacZ4787*(::rrnB-3), λ^-, *rph-1*, *Δ(rhaD-rhaB)568*, *hsdR514*, pKD46	CGSC

pCP20	F-, *Δ(argF-lac)169*, φ80d*lacZ58*(M15), *glnX44*(AS), λ^{-}, *rfbC1*, *gyrA96*(NalR), *recA1*, *endA1*, *spoT1*, *thiE1*, *hsdR17*, pCP20	CGSC
pSUB11	F-, *Δ(araD-araB)567*, *ΔlacZ4787*(::rrnB-3), *Δ(phoB-phoR)580*, λ^{-}, *galU95*, *ΔuidA3::pir*$^{+}$, *recA1*, *endA9*(del-ins)*::FRT*, *rph-1*, *Δ(rhaD-rhaB)568*, *hsdR514,* pSUB11.	Gillian’s lab.
