# Supplementary material for: Repression of YdaS Toxin Is Mediated by Transcriptional Repressor RacR in the Cryptic rac Prophage of Escherichia coli K-12
Source: mSphere. 2017 Nov 22;2(6):e00392-17. doi: 10.1128/mSphere.00392-17 (PMC5700373; doi:10.1128/mSphere.00392-17)
Supplement: TABLE S2 [file sph005172392st7.pdf]

Supplementary Table2: Primers used in this study

**Primer Description**	**Sequence 5' -----> 3'**
kilR_pkd13_F	ACCGCATCAACAAAGTTCATTTGTAAAAATGGAGATAATTgtgtaggctggagctgcttcg
kilR_pkd13_R	TTTTTGCAAAGGTGGTAAGCACATTTTATTTTCTTAGTCAattccggggatccgtcgacc
racR_pkd13_F	GGGATTGCCTAATGTAATGCGCATAGGAGAATATTAAGCAgtgtaggctggagctgcttcg
racR_pkd13_R	AATAACGGAATCCAGGAGTTTTCCGTCAGACCATATAAGTattccggggatccgtcgacc
ydaS_pkd13_F	GCGTCGCCTAATATTTCTGTGTGTTTTTGGAGTTCATTCGAgtgtaggctggagctgcttcg
ydaS_pkd13_R	CTCATGCTTGATTTTCATGAATCATTTGCCTCTTGATGTTattccggggatccgtcgacc
ydaT_pkd13_F	CATTTGATCATACCTGAAACATCAAGAGGCAAATGATTCAgtgtaggctggagctgcttcg
ydaT_pkd13_R	GTGGCTTAGAATAAGCACAAACAGCATGGAAACTTTTGCattccggggatccgtcgacc
intR ttcC F	ATTTCAGTTCTCTGGTACTAAATGGGGCAAATTGGGGGCAAACTTTGCAAgtgtaggctggagctgcttcg
intR ttcC R	GCATGACGCATACTCTTCTGATGCCATATAACGAATTGAGTCGCTTTTAAattccggggatccgtcgacc
kilR ydaV F	TTCAACGTCTTTTTTGCAAAGGTGGTAAGCACATTTTATTTTCTTAGTCAgtgtaggctggagctgcttcg
kilR ydaV R	CTTTCAGTGCGTCAAAAACAGTCTCCATTAAATTTTTCTCCCGGTAAAAAattccggggatccgtcgacc
ralR ynaK F	TTGTCCAGTTAGTAGGAGTGCCACCTTCCTTTTCAATAGTGGCGGTAATTgtgtaggctggagctgcttcg
ralR ynaK R	TTTTCTCAATGTGGCGACGGATTAATGCATTACGGGAGCGATACTGATCGattccggggatccgtcgacc
recT trkG F	TCTCATAAAAAATATTTCAAGTTGGCGGTGCATTACACCGCCAGGCTGAAgtgtaggctggagctgcttcg
recT trkG R	ATGAGTGAGTCAACATAATATTAAACTCACAATTATAAATCAGCCATATAattccggggatccgtcgacc
sieB ydaU F	CGAGAGCTTGTGTTAACATTTCAATACCCTTACAGTTGAGAGTTATTGATgtgtaggctggagctgcttcg
sieB ydaU R	CTGCGGATACGTTCAAGAACATCGCCTGTCGCAATATTTTTCATGGTCAGattccggggatccgtcgacc

ydaF ydaT F	TCCCATTTTATGAAGTTATTCTGGAACAGCAGGAGTAGACGTTTTAATCGgtgtaggctggagctgcttcg
ydaF ydaT R	GATGCTGCCCGGTGGCTTAGAATAAGCACAAACAGCATGGAAACTTTTGCattccggggatccgtcgacc
ydaQ ynaA F	TGCGAATGTATCTACCTCTAATCTCGACACCTGTTGGTAATTTAGACATAgtgtaggctggagctgcttcg
ydaQ ynaA R	CATCGCATACGCGCTGAACCATTCATTCACGCGCACAGACGGCCCCACCAattccggggatccgtcgacc
racR_ydaS_IGR_F	CTCATGCTTGATTTTCATGAATCATTTGCCTCTTGATGTTgtgtaggctggagctgcttcg
racR_ydaS_IGR_R	AATAACGGAATCCAGGAGTTTTCCGTCAGACCATATAAGTattccggggatccgtcgacc
Tag RacR 3X F	AAGCTCTTGAATCTGAACGGAAAAGCCAGAACATCACAAAAACTGGAACTgactacaaagaccatgacgg
Tag RacR 3X R	GGGGGGGTTAAATAACGGAATCCAGGAGTTTTCCGTCAGACCATATAAGTcatatgaatatcctccttag
Tag ydaS 3X F	TGTCAGTGAAGCAACTAAATGACAGTAACAAATCCTCATTTGATCATACCgactacaaagaccatgacgg
Tag ydaS 3X R	ATTCGATGTGCTCATGCTTGATTTTCATGAATCATTTGCCTCTTGATGTTcatatgaatatcctccttag
ydaT _F_EcoRI	CCGGAATTC ATGAAAATCAAGCATGAGCACATCG
ydaT_R_SalI	CGGCGGGTCGACTTAATGAACAATGACAGAATCGTC
ydaS _F_EcoRI	CCGGAATTCATGAAAAAAGAGAACTATTCATTCAAGC
ydaS _R_SalI	CGGCGGGTCGACTCAGGTATGATCAAATGAGGATTTG
racR_F_NdeI	CGCCATATGCTTAGTGGTAAAGAC
racR_R_XhoI	CCGCTCGAGAGTTCCAGTTTTTGTGAT
TSA_189_FW	CCGCTCGAGGATTTGACGGATCCCGATG
TSA_189_RV	AAAACTGCAGTCAGGTATGATCAAATGAGG
TSA_whole_FW	CCGCTCGAGTTAAGTTCCAGTTTTTGTG
TSA-whole_RV	AAAACTGCAGTCAGGTATGATCAAATGAGG
IGR_RT_FW	CGAATGAACTCCAAAAACACACAGA
IGR_RT_RV	TCCTATGCGCATTACATTAGGCA
wza_RT_FW	ATGATGAAATCCAAAATGAAATTGATGCC
wza_RT_RV	CATTTTGTCGAGATCGAAATCAGCGTC
EMSA_123bp_F	CGAATGAACTCCAAAAACACACAGA
EMSA_123bp_R	TGCTTAATATTCTCCTA

EMSA_49bp_F	CGAATGAACTCCAAAAACACACAGA
EMSA_49bp_R	TTGCGTTAGGCGTCGCCTAATA
ydaS_Prom_XhoI_F	CCGCTCGAGTGCTTAATATTCTCCTATGC
ydaS_Prom_R_BamHI	CGCGGATCCCGAATGAACTCCAAAAACA
