# Supplementary material for: Repression of YdaS Toxin Is Mediated by Transcriptional Repressor RacR in the Cryptic rac Prophage of Escherichia coli K-12
Source: mSphere. 2017 Nov 22;2(6):e00392-17. doi: 10.1128/mSphere.00392-17 (PMC5700373; doi:10.1128/mSphere.00392-17)
Supplement: TABLE S3 [file sph005172392st8.pdf]

Supplementary Table 3: List of accession numbers of RNA seq data used for calculating RPKM

GSE63858	Ribosome profiling of E. coli K-12 MG1655 MOPS rich media with 0.2% glucose
GSE72899	Clarifying the translational pausing landscape in bacteria by ribosome profiling (1)
GSE72899	COLOMBOS v2.0: an ever expanding collection of bacterial expression compendia (2)
E-MTAB-2802	Comprehensive Mapping of the *Escherichia coli* Flagellar Regulatory Network (3)
GSE54901	Deciphering Fur transcriptional regulatory network highlights its complex role (4)
GSE66482	Decoding genome-wide GadEWX-transcriptional regulatory networks reveals multifaceted cellular responses to acid stress in *Escherichia coli (5)*
GSE46740	Genome-scale reconstruction of the sigma factor network in *Escherichia coli*: topology and functional states (6)
E-MTAB-2903	Identification of bacterial sRNA regulatory targets using ribosome profiling (7)
GSE55199	Global Transcriptional Start Site Mapping Using Differential RNA Sequencing Reveals Novel Antisense RNAs in *Escherichia coli (8)*
GSE41940	Rho and NusG suppress pervasive antisense transcription in *Escherichia coli(9)*
E-MTAB-4240	SuhB Associates with Nus Factors To Facilitate 30S Ribosome Biogenesis in

	Escherichia coli(10)
GSE69856	The ribonuclease polynucleotide phosphorylase can interact with small regulatory RNAs in both protective and degradative modes (11)
GSE82343	Modulation of global transcriptional regulatory networks as a strategy for increasing kanamycin resistance of EF-G mutants
GSE40313	Genomic analysis reveals epistatic silencing of "expensive" genes in Escherichia coli K-12 (12)
GSE104504	Transcriptome data for wild type , *Δfis* , *Δcya* mutants in Early Exponential, Mid Exponential growth phase generated in-house (unpublished)

BIBLIOGRAPHY

1. **Mohammad F**, **Woolstenhulme CJ**, **Green R**, **Buskirk AR**. 2016. Cell Rep **14**:686–694.

2. **Meysman P**, **Sonego P**, **Bianco L**, **Fu Q**, **Ledezma-Tejeida D**, **Gama-Castro S**, **Liebens V**, **Michiels J**, **Laukens K**, **Marchal K**, **Collado-Vides J**, **Engelen K**. 2014. Nucleic Acids Res **42**:D649–D653.

3. **Fitzgerald DM**, **Bonocora RP**, **Wade JT**. 2014. PLoS Genet **10**:e1004649.

4. **Seo SW**, **Kim D**, **Latif H**, **O'Brien EJ**, **Szubin R**, **Palsson BO**. 2014. Nat Commun **5**:4910.

5. **Seo SW**, **Kim D**, **O'Brien EJ**, **Szubin R**, **Palsson BO**. 2015. Nat Commun **6**:7970.

6. **Cho B-K**, **Kim D**, **Knight EM**, **Zengler K**, **Palsson BO**. 2014. BMC Biol **12**:4.

7. **Wang J**, **Rennie W**, **Liu C**, **Carmack CS**, **Prévost K**, **Caron M-P**, **Massé E**, **Ding Y**, **Wade JT**. 2015. Nucleic Acids Res gkv1158.

8. **Thomason MK**, **Bischler T**, **Eisenbart SK**, **Förstner KU**, **Zhang A**, **Herbig A**, **Nieselt K**, **Sharma CM**, **Storz G**. 2015. J Bacteriol **197**:18–28.

9. **Peters JM**, **Mooney RA**, **Grass JA**, **Jessen ED**, **Tran F**, **Landick R**. 2012.Genes Dev **26**:2621–2633.

10. **Singh N**, **Bubunenko M**, **Smith C**, **Abbott DM**, **Stringer AM**, **Shi R**, **Court DL**, **Wade JT**. 2016. mBio **7**:e00114-16.

11. **Bandyra KJ**, **Sinha D**, **Syrjanen J**, **Luisi BF**, **De Lay NR**. 2016. RNA **22**:360–372.

12. **Srinivasan R**, **Chandraprakash D**, **Krishnamurthi R**, **Singh P**, **Scolari VF**,

Krishna S, **Seshasayee ASN**. 2013. Mol Biosyst **9**:2021.
